# Assessing Preference Shift and Effects on Patient Knowledge and Decisional Conflict: Cross-Sectional Study of an Interactive Prostate-Specific Antigen Test Patient Decision Aid

**DOI:** 10.2196/11102

**Published:** 2018-11-21

**Authors:** Peter Scalia, Glyn Elwyn, Jan Kremer, Marjan Faber, Marie-Anne Durand

**Affiliations:** 1 The Dartmouth Institute for Health Policy and Clinical Practice Dartmouth College Lebanon, NH United States; 2 Radboud Institute for Health Sciences Scientific Institute for Quality of Healthcare Radboud university medical center Nijmegen Netherlands

**Keywords:** decision aids, decision making, prostate-specific antigen, conflict, patient preference, prostate screening

## Abstract

**Background:**

Randomized trials of Web-based decision aids for prostate-specific antigen (PSA) testing indicate that these interventions improve knowledge and reduce decisional conflict. However, we do not know about these tools’ impact on people who spontaneously use a PSA testing patient decision aid on the internet.

**Objective:**

The objectives of this study were to (1) determine the impact of the Web-based PSA Option Grid patient decision aid on preference shift, knowledge, and decisional conflict; (2) identify which frequently asked questions (FAQs) are associated with preference shift; and (3) explore the possible relationships between these outcomes.

**Methods:**

Data were collected between January 1, 2016, and December 30, 2017. Users who accessed the Web-based, interactive PSA Option Grid were provided with 3 options: have a PSA test, no PSA test, or unsure. Users first declared their initial preference and then completed 5 knowledge questions and a 4-item (yes or no) validated decisional conflict scale (Sure of myself, Understand information, Risk-benefit ratio, Encouragement; SURE). Next, users were presented with 10 FAQs and asked to identify their preference for each question based on the information provided. At the end, users declared their final preference and completed the same knowledge and decisional conflict questions. Paired sample *t* tests were employed to compare before and after knowledge and decisional conflict scores. A multinomial regression analysis was performed to determine which FAQs were associated with a shift in screening preference.

**Results:**

Of all the people who accessed the PSA Option Grid, 39.8% (186/467) completed the interactive journey and associated surveys. After excluding 22 female users, we analyzed 164 responses. At completion, users shifted their preference to “not having the PSA test” (43/164, 26.2%, vs 117/164, 71.3%; *P*<.001), had higher levels of knowledge (112/164, 68.3%, vs 146/164, 89.0%; *P*<.001), and lower decisional conflict (94/164, 57.3%, vs 18/164, 11.0%; *P*<.001). There were 3 FAQs associated with preference shift: “What does the test involve?” “If my PSA level is high, what are the chances that I have prostate cancer?” and “What are the risks?” We did not find any relationship between knowledge, decisional conflict, and preference shift.

**Conclusions:**

Unprompted use of the interactive PSA Option Grid leads to preference shift, increased knowledge, and reduced decisional conflict, which confirms the ability of these tools to influence decision making, even when used outside clinical encounters.

## Introduction

Randomized trials of Web-based decision aids for the prostate-specific antigen (PSA) screening test have indicated that these tools increase user knowledge, reduce decisional conflict, and reduce interest in having the test in controlled contexts, where users are recruited to use the intervention [1,2]. However, what can we say about users who choose to use Web-based decision aids independent of any study recruitment? Does spontaneous use of a PSA tool have an effect on users’ knowledge and decisional conflict and shift their screening preferences?

A recent systematic review of 13 randomized trials assessed the effectiveness of decision aids for decision making in prostate cancer testing [3]. Regardless of the mode of delivery (ie, paper-based, Web-based, or video) the majority of decision aids improved patient knowledge of the PSA screening test and mitigated decisional conflict [3]. An example of a Web-based intervention that induced these positive outcomes is Prosdex, which also lowered the intention to undergo testing by 18% in comparison with participants in the control group who did not receive any decision support intervention [1]. Furthermore, a randomized trial found an almost 10% reduction in PSA screening and a 30% increase in preference for “watchful waiting” for those who used a Web-based decision aid compared to those who viewed public websites [4]. Web-based PSA tools that are tailored to individual participants increased knowledge levels among African American men, improved decision quality, and decreased levels of decision regret at follow-up [5-7].

A Web-based PSA decision aid—“PSA test: yes or no?” Option Grid—is a platform for men seeking information on the Web. It is designed for independent use. Individuals (presumably men) find this tool, independent of any invitation or promotion. This tool provides evidence-based information on the risks and pros and cons of the PSA screening test to help users make a decision that aligns with their preference [8]. The tool assesses user knowledge, level of decisional conflict, and preference before and after viewing the information. A prior study among 82 participants shows that users of the PSA Option Grid tend to become more risk averse, shifting their preference to “not having the test” after viewing risk information associated with the screening test and prostate biopsies [9]. This indicates a real-world impact on screening preference for users who spontaneously use this tool [9].

In a recent study, researchers used Google Analytics to track usage data for users of a Web-based decision aid for early-stage prostate cancer to determine if the tool was helpful and if users would recommend it to others. Although this study analyzed data from an unsolicited sample to determine “real-world” impact, they did not assess outcomes pre- and post decision aid use [10]. As far as can be determined, there has been no assessment of the direct impact of using a Web-based PSA screening decision aid on specific outcomes like preference shift, knowledge, or decisional conflict without actively recruiting or providing incentives to users in a research context.

The aims of this study were to: (1) determine the impact of the Web-based PSA Option Grid patient decision aids on preference shift, knowledge, and decisional conflict; (2) identify which frequently asked questions (FAQs) are associated with preference shift; and (3) explore the possible relationships between these outcomes.

## Methods

### Design

We conducted an analysis of data from a longitudinal sample derived from the Option Grid website of users who searched for and used the Web-based “PSA test: yes or no?” Option Grid, independent of any invitation. We assessed user preferences regarding PSA screening. We also measured levels of knowledge and decisional conflict before and after using the intervention. Ethical approval for this study was received from the Dartmouth College Committee for the Protection of Human Subjects (STUDY00030776).

### Participants

Data from users of the “PSA test: yes or no?” Option Grid collected between January 1, 2016, and December 30, 2017, were eligible for inclusion. Data were excluded if the user exited the Option Grid website prior to completing the entire interactive process or if the user self-identified as female.

### Intervention

Based on the 2017 Cochrane systematic review definition of a patient decision aid—“decision aids are intended to provide information and to promote self-help in the treatment decision-making process, which enables the patient to more actively participate in this process, if this is his or her preference”—we identify Option Grid as a patient decision aid [11]. Option Grid is available in static (PDF) and interactive (Option Grid interactive journey) formats. Both the paper-based and interactive versions of the “PSA test: yes or no?” Option Grid were freely available on the optiongrid.org website until March 2018. Users searched for and used the tools independent of any invitation. The tool was not promoted at any time during the study period. The interactive version of Option Grid was intended for independent use, but the information provided could have been used to facilitate a more collaborative discussion with a physician.

On the Option Grid website, users could have searched for the PSA interactive Option Grid using the keyword function or found it on a list of topics they could have browsed through. Once on the PSA Option Grid webpage, users had the option of viewing the PDF version of the PSA Option Grid or starting the “interactive journey.” The same information is presented in both versions except the interactive journey presents the information in a sequential interactive method. If the journey was selected, users provided their demographic information such as their age group, gender, ethnicity, and geographic location. Before proceeding, users identified the strength of their preference, their level of decisional conflict, and their level of knowledge. Next, 10 FAQs, always presented in the same order, provided users with evidence-based information on the PSA test (ie, described the test and indicated the chances of having prostate cancer in their lifetime, the significance of having a normal or high PSA level, survival risk, and the advantages and risks associated with the PSA test), and the risks and side effects associated with prostate biopsies and prostate cancer treatments. Multimedia Appendix 1 illustrates the interface that the user encountered for the first FAQ of the tool. To complete the interactive journey, users identified their final preference and the strength of that preference and completed the same SURE survey and knowledge questionnaire post-PSA Option Grid use.

### Outcome Measures

The “self-check” knowledge measure contained 5 items that require a true or false response (Multimedia Appendix 2). The questions were developed in relation to some of the information embedded in the interactive PSA Option Grid. Those questions helped us determine if the user understood the content and learned new information during the interactive journey. Users filled out their responses before and after completing the journey.

Légaré et al developed a short 4-item decisional conflict measure known as SURE (Sure of myself; Understand information; Risk-benefit ratio; Encouragement), in which the user responded yes or no to each of the 4 questions (Multimedia Appendix 3) [12]. The 4 items are based on the 16-item decisional conflict scale and the Ottawa Decision Support Framework. The reliability and validity of SURE was first assessed with French-speaking pregnant women considering prenatal screening for Down syndrome and with over 1000 English-speaking patients in rural New England who were referred to watch condition-specific video decision aids [12]. SURE was found to be a reliable and valid measure to “detect clinically significant decisional conflict” in both groups [12,13]. Results of a secondary analysis of a clustered randomized trial supported the conclusions using a primary care sample: SURE showed “adequate psychometric properties” [13].

We also collected user preference data before and after reading the information associated with each FAQ in the interactive Option Grid (Multimedia Appendix 4).

### Data Collection and Analysis

A database stored all responses provided by the user throughout the entire interactive journey; we only analyzed data from users of the interactive version. This included their responses to the knowledge questions, SURE survey, and their preferences pre-and post-Option Grid use.

The 3 preference options were represented in the dataset as 0=having the PSA test, 1=not having the PSA test, and 2=I am not sure. The McNemar test was used to determine if users significantly shifted their preference after completing the interactive journey in comparison with their initial preference prior to viewing the information. Chi-square tests were performed to explore possible relationships between knowledge, decisional conflict, and preference shift.

We conducted a multinomial regression analysis to determine which FAQs were associated with preference shift. We created a dependent variable with 4 categories: 0=“having a PSA test” shifted to “not having a PSA test,” 1=“not having a PSA test” shifted to “having a PSA test,” 2=“not having a PSA test” preference retained, and 3=“having a PSA test” preference retained (reference category). The FAQs represented nominal independent variables and were inserted as factors in the model. Due to the fact that multiple treatment options are being compared for each FAQ, we decided that FAQs with a *P* value of ≤.02 would be considered statistically significant in terms of shifting user preference [9].

Users received a score from 0 to 5 (a perfect score) on the “self-check” knowledge questionnaires. This score was recorded as a continuous variable in the database. The pre- and post knowledge scores were used in a paired sample *t* test to determine whether knowledge significantly increased after Option Grid use. The dataset also contained the user’s responses (0=no, 1=yes) to each SURE survey item before and after completing the interactive Option Grid. A perfect score indicated that the user was not experiencing clinically significant decisional conflict. A score of ≤3 meant that the user was experiencing clinically significant decisional conflict [13]. A paired sample *t* test was employed to compare the total pre-and postdecisional conflict scores.

## Results

### Participant Sample

A total of 467 users accessed the Option Grid website and began using the Web-based, interactive PSA decision aid. However, only 186 users completed the entire interactive journey. Of the 186 completed “journeys” (attrition rate of 60.2%, 281/467), 22 users self-identified as female, leaving a sample of 164 users. The majority of 281 users who dropped out either did so after viewing the first FAQ (118/281, 42.0%) or at the midway point of the journey (66/281, 23.5%). Over half (88/164, 53.7%) of users indicated that they were between the ages of 45-64 years, and over 70.1% (115/164) of the sample self-identified as white or not Hispanic or Latino. The majority (87/164, 53.0%) of the sample resided in North America. See Table 1 for details.

### Preference Shift

**Figure 1:**
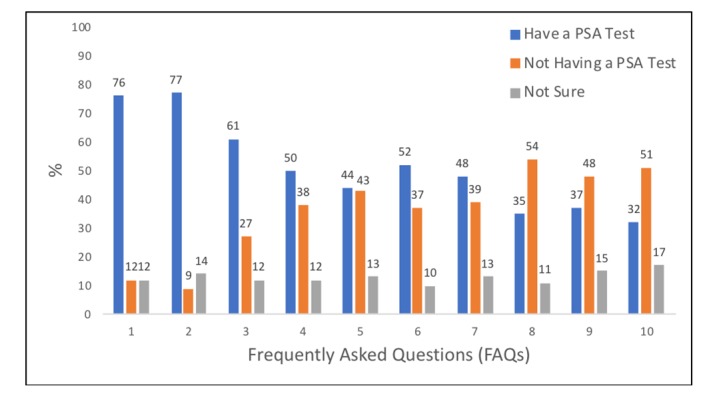
Percentage of users who declared their preference for each FAQ: have a prostate-specific antigen (PSA) test, not having a PSA test, or not sure.

Prior to being presented with the FAQs, 73.8% (121/164) users selected “having the PSA test” as their initial preference. After completing the interactive journey, 28.7% (47/164) users indicated that they preferred having the PSA test—a decrease of 45.1% (74/164). The number of users who preferred “not having the PSA test” increased from 43 users pre-FAQ to 117 users post-FAQ. Overall, a significant preference shift (*P*<.001) to “not having a PSA test” occurred after viewing the information embedded in the interactive tool. Figure 1 illustrates the decrease in the number of users who selected “having a PSA test” for each FAQ.

**Table 1:** User characteristics for the Web-based, interactive “prostate-specific antigen test: yes or no?” Option Grid.

Characteristic		n (%)
**Age in years**		
	18-24	5 (3.0)
	25-44	29 (17.7)
	45-64	88 (53.7)
	>65	42 (25.6)
**Ethnicity**		
	Hispanic or Latino	15 (9.1)
	Not Hispanic or Latino	115 (70.1)
	Not identified	34 (20.7)
**Race**		
	White	130 (79.3)
	Black or African American	11 (6.7)
	Asian	12 (7.3)
	Native Hawaiian or other Pacific Islander	1 (0.6)
	American Indian or Alaska Native	5 (3.0)
	Other or not identified	5 (3.0)
**Geographic region**		
	North America	87 (53.0)
	South America	8 (4.9)
	Europe	59 (36.0)
	Africa	1 (0.6)
	Asia	5 (3.0)
	Australia	4 (2.4)

There were 3 FAQs associated with a preference shift from “having the PSA test” to “not having the PSA test,” namely, FAQ1, “What does the test involve?” (*P*=.002); FAQ 3, “If my PSA level is high, what are the chances that I have prostate cancer?” (*P*=.01); and FAQ 7, “What are the risks?” (*P*=.01; Table 2). The majority of users in the sample selected “having a PSA test” for FAQ 1, which informed them that the PSA is a blood test that measures the antigen level in the blood from the prostate gland; no information was provided for the “not having a PSA test” option. A slight increase in PSA preference occurred for FAQ 2, where users were presented with the same risk information for both options: 15% of men will develop prostate cancer in their lifetime. Numbers significantly declined for preference of the PSA test at FAQs 3 and 4. When users were presented with the fact that 30% of men with a high PSA level have prostate cancer (FAQ 3), albeit inflammation and infection also increase levels, they shifted their preference to “not having the PSA test.” Preference for this option continued with FAQ 4, which stated that 15% of men with a normal PSA have prostate cancer. More users opted for “not having the test” at FAQ 5, which stated that only 0.6% of men who do not have a PSA test die from prostate cancer. FAQ 6 represented an inflection point because this was the only question that reversed the trend (albeit not significantly) of preferences shifting to “having the test.” FAQ 6 indicated that 33% of prostate cancers are aggressive, and a small number will benefit from early treatment. FAQ 7 significantly shifted preference. It informed users that the PSA test cannot identify an aggressive form of prostate cancer and that more tests (biopsies) would be needed. The number of users who preferred having the screening test continued to decline after this question until it hit the lowest point at the last question when only 31.7% (52/164) of the sample preferred having the PSA test.

### Knowledge

Before viewing the FAQs, 68.3% (112/164) users achieved a perfect score compared with the 89.0% (146/164) users who achieved a perfect knowledge score after viewing the FAQs. The mean post-FAQ knowledge score was 4.88 (SD 0.36) compared with the pre-FAQ knowledge score of 4.64 (SD 0.56). Overall, there was a statistically significant knowledge increase after viewing the Web-based, interactive PSA Option Grid FAQs (*t*_163_=−6.70, *P*<.001).

### Decisional Conflict

Before reviewing the FAQs, 89.0% (146/164) users answered “no” to at least 1 of the 4 SURE survey items, indicating decisional conflict. After completing the Option Grid interactive journey, decisional conflict decreased to 42.7% (70/164) users. Overall, the decisional conflict score pre-FAQs was 1.49 (SD 1.38) and post-FAQs was 3.24 (SD 1.03). A statistically significant decisional conflict reduction occurred after viewing the Web-based, interactive PSA Option Grid (*t*_163_=−15.234, *P*<.001). The percentages of users who selected yes for each SURE item are listed in Table 3.

### Relationship Between Knowledge, Decisional Conflict, and Preference

Analyses indicated no association or relationship between preference shift and increased knowledge levels (*P*=.45) or between reduced decisional conflict and preference shift (*P*=.29). Furthermore, no relationship was established between increased knowledge and reduced decisional conflict (*P*=.85).

**Table 2:** Frequently asked questions (FAQs) associated with preference shift for the interactive prostate-specific antigen (PSA) Option Grid decision aid based on the multinomial regression analysis.

Variable^a^		“Having a PSA” shifted to “not having a PSA” (n=76), OR^b^ (95% CI)	“Not having a PSA” shifted to “having a PSA” (n=2), OR (95% CI)	“Not having a PSA” preference retained (n=41), OR (95% CI)	*P* value^c^
**FAQ 1: What does the test involve?**					.002^e^
	Not sure	1.00	1.00	1.00	
	Have a PSA test	0.48 (0.07-3.09)	205 (0.00-0.00)^d^	9.7×10^6^ (0.00-0.00)^d^
	Not have PSA test	5.9×10^5^ (0.00-0.00)^d^	0.00 (0.00-0.00)^d^	7.0×10^13^ (0.00-0.00)^d^
**FAQ 2: What are my chances of having prostate cancer in my lifetime?**					.94
	Not sure	1.00	1.00	1.00	
	Have a PSA test	0.40 (0.04-3.73)	0.00 (0.00-0.00)^d^	0.27 (0.02-4.09)
	Not have PSA test	0.12 (0.00-0.00)^d^	3.89 (0.00-0.00)^d^	0.03 (0.00-0.00)^d^
**FAQ 3: If my PSA level is high, what are the chances that I have prostate cancer?**					.01^e^
	Not sure	1.00	1.00	1.00	
	Have a PSA test	1.02 (0.08-13.63)	236 (0.00-0.00)^d^	0.07 (0.00-1.81)
	Not have PSA Test	0.71 (0.04-13.73)	0.00 (0.00-0.00)^d^	0.26 (0.01-8.23)
**FAQ 4: If my PSA level is normal, can I be sure that I don’t have prostate cancer?**					.26
	Not sure	1.00	1.00	1.00	
	Have a PSA test	0.11 (0.01-1.33)	2.81×10^10^ (0.00-0.00)^d^	0.57 (0.02-14.53)
	Not have PSA test	0.33 (0.02-4.71)	94.79 (0.00-0.00)^d^	1.13 (0.04-30.95)
**FAQ 5: Will getting the PSA test lower my chance of dying from prostate cancer?**					.07
	Not sure	1.00	1.00	1.00	
	Have a PSA test	13.43 (0.73-246)	2.06×10^11^ (0.00-0.00)^d^	114.96 (0.78-169.02)
	Not have PSA test	17.69 (0.88-353)	0.00 (0.00-0.00)^d^	223.42 (1.51-330.33)
**FAQ 6: What are the advantages?**					.09
	Not sure	1.00	1.00	1.00	
	Have a PSA test	1.60 (0.09-30.35)	0.00 (0.00-0.00)^d^	1.66 (0.05-51.22)
	Not have PSA test	16.23 (0.70-377)	0.25 (0.00-0.00)^d^	10.72 (0.31-373.95)
**FAQ 7: What are the risks?**					.01^e^
	Not sure	1.00	1.00	1.00	
	Have a PSA test	0.26 (0.01-11.67)	0.00 (0.00-0.00)^d^	0.05 (0.00-2.95)
	Not have PSA test	0.76 (0.02-39.09)	0.00 (0.00-0.00)^d^	0.26 (0.00-17.42)
**FAQ 8: What risks are associated with a prostate biopsy?**					.99
	Not sure	1.00	1.00	1.00	
	Have a PSA test	2.75 (0.05-154)	0.00 (0.00-0.00)^d^	0.98 (0.01-167.57)
	Not have PSA test	4.33 (0.07-255)	7.64 (7.64-7.64)^d^	1.37 (0.01-237.35)
**FAQ 9: What other side effects can I expect from a prostate biopsy?**					.89
	Not sure	1.00	1.00	1.00	
	Have a PSA test	0.25 (0.01-4.33)	0.00 (0.00-0.00)^d^	0.43 (0.01-15.63)
	Not have PSA test	0.24 (0.01-4.27)	0.00 (0.00-0.00)^d^	0.53 (0.02-18.50)
**FAQ 10: What are the risks associated with prostate cancer treatment?**					.61
	Not sure	1.00	1.00	1.00	
	Have a PSA test	0.23 (0.03-1.88)	0.00 (0.00-0.00)^d^	0.50 (0.03-7.45)
	Not have PSA test	0.42 (0.05-3.53)	0.00 (0.00-0.00)^d^	0.39 (0.03-5.95)

^a^Reference category: “having a PSA test” preference retained.

^b^OR: odds ratio.

^c^Estimated *P* value for the association between FAQs and preference shift.

^d^The cell sample size was too small; thus, we could not compute OR or CI.

^e^Significantly shifted screening preference.

**Table 3:** The proportion of users who responded “yes” to each item on the Sure of myself, Understand information, Risk-benefit ratio, Encouragement (SURE) decisional conflict survey before and after viewing the information embedded in the interactive prostate-specific antigen Option Grid.

SURE item	Yes, n (%)	
	Pre	Post
Do you feel sure about the best choice for you?	55 (33.5)	110 (67.1)
Do you know the benefits and risks of each option?	52 (31.7)	156 (95.1)
Are you clear about which benefits and risks matter most to you?	63 (38.4)	136 (82.9)
Do you have enough support and advice to make a choice?	74 (45.1)	129 (78.7)

## Discussion

### Principal Findings

The Web-based interactive PSA Option Grid decision aid shifted preference toward not having the screening test, increased user knowledge, and reduced decisional conflict. In particular, there were 3 elements of information that induced a shift. First, the description of the PSA test—a blood test that measures the antigen level in the blood from the prostate—was associated with the preference of having the PSA test. Second, FAQ 3 (stating that 30% of men with a high PSA level have prostate cancer, but that inflammation and infection can also increase PSA levels) shifted user preferences to declining the screening test. Lastly, the risks of having the PSA test were presented at FAQ 7, which represents a significant juncture in the “journey” in terms of shifting user preference to not having the PSA test. FAQ 7 stated that it is not possible to know whether a cancer is aggressive with the PSA test alone; a high PSA level means that one would need more tests like biopsies, and biopsies carry risks. No relationships were established between knowledge, decisional conflict, and preference shift.

The main strength of our study is that we obtained information from a self-selected sample of participants who freely accessed the Web-based intervention to better understand whether the effect of using a Web-based tool is replicated in a naturalistic setting (ie, outside of a controlled, incentivized research context). However, we know that a self-selected sample of individuals who access Web-based health information is likely to have a higher computer literacy and educational attainment, which means that we may not have had a representative sample of the greater population. Increasing the sample size and randomizing the FAQs would strengthen the study findings. We recognize that having a more diverse sample may have influenced our findings. Only 6.7% (11/164) participants of our sample identified as African American people, and we know that this patient population is considered to be at high risk for prostate cancer [14]. Further, we were unable to determine whether users were health care professionals or actual patients. Lastly, it is important to note that following data analysis, the interactive PSA Option Grid decision aid (in the Web-based format used for this study) was removed from the Web in March 2018 and is no longer available for public use.

A paucity of data exists on the outcomes associated with the use of Web-based PSA decision aids for individuals spontaneously searching the internet for information. Our study shows that even for a self-selected sample, a Web-based tool increased knowledge and reduced decisional conflict. Our previous work indicated that FAQs 1, 3, and 8 shifted user preferences to not having the PSA screening test. In this study, FAQs 1, 3, and 7 shifted preference in the same direction, confirming that risk information (FAQs 7 and 8 both discuss risk) may be the *active ingredient* in the PSA Option Grid responsible for the shift [9]. FAQs 7 and 8 discuss the risk of the PSA test and the risk of the prostate biopsy, respectively. Thus, we can infer that men value risk information in their decision making. Our data are also consistent with previous studies suggesting that men who used Web-based decision aids reported higher knowledge and lower decisional conflict and were less likely to want prostate cancer screening [1,2,15,16]. The preference not to undergo screening aligns with the Agency for Healthcare Research and Quality’s recommendation, which indicates that the “benefits of PSA-based screening for prostate cancer do not outweigh the harms” [17].

Our study showed no relationship between knowledge, decisional conflict, and preference. This differs from Evans et al’s randomized trial that showed a link between increased knowledge and a less favorable attitude toward testing [1]. Although we did not test attitude, we still did not see an association between increased knowledge and preference shift. Rubel et al used a Solomon 4-group design to demonstrate that increased knowledge was related to reduced decisional conflict for those using a prostate cancer screening decision aid [18]. Based on our data, we can infer that men who spontaneously used the Option Grid already had high levels of knowledge to begin with, despite being conflicted about their screening preference. Further investigation is required to better understand the potential associations between these outcomes. We did not collect data on the final screening decision of the user; thus, more research is needed to understand the actual effect of using interactive decision aids on the quality of the real-world decision-making process.

In light of the high attrition rate observed in this study, future work should focus on creating or modifying Web-based patient decision aids to reduce the burden on the user. Many men search the internet for credible health information, but the high attrition rate in our study indicates that interest or engagement is impacted by the time it takes to complete the Option Grid interactive journey. For example, an observational Web-log analysis showed that the mean total time spent on a Web-based decision aid is 20 minutes [19]. Evidently, these tools, which rarely undergo extensive usability testing, can be made easier to use [20]. Furthermore, research should focus on minimizing the “digital divide” [21]. Men who use these tools tend to be white people, highly educated, and reasonably computer literate, with internet access [21,22]. Men exhibiting these characteristics have a significant advantage in terms of access to health information. We need to better understand how to reach men across socioeconomic strata and how to create Web-based tools that are suitable to all demographics, health literacy levels, and computer literacy levels.

### Conclusion

The Web-based PSA Option Grid decision aid enabled users to increase their level of knowledge while reducing decisional conflict. The risk information embedded in the tool shifted preference away from having the screening test. Efforts should be made to increase access to evidence-based information for men in all socioeconomic categories. This would lead men to be more informed when communicating with their clinician and would help them make a decision that aligns with their preference.

## Appendix

Multimedia Appendix 1 The interface the user encountered for the first frequently asked question of the Web-based PSA Option Grid patient decision aid.

Multimedia Appendix 2 The "self-check" knowledge questionnaire that users encountered before, and after completing the interactive journey.

Multimedia Appendix 3 The SURE survey presented to users before and after completing the interactive journey to measure decisional conflict.

Multimedia Appendix 4 Users identify their preference pre-and-post interactive journey.

## Abbreviations

FAQ: frequently asked question
PSA: prostate-specific antigen
SURE: Sure of myself, Understand information, Risk-benefit ratio, Encouragement

## References

[1] Evans R, Joseph-Williams N, Edwards A, Newcombe RG, Wright P, Kinnersley P, Griffiths J, Jones M, Williams J, Grol R, Elwyn G (2010). Supporting informed decision making for prostate specific antigen (PSA) testing on the web: an online randomized controlled trial. J Med Internet Res.

[2] Taylor KL, Williams RM, Davis K, Luta G, Penek S, Barry S, Kelly S, Tomko C, Schwartz M, Krist AH, Woolf SH, Fishman MB, Cole C, Miller E (2013). Decision making in prostate cancer screening using decision aids vs usual care: a randomized clinical trial. JAMA Intern Med.

[3] Ilic D, Jammal W, Chiarelli P, Gardiner RA, Hughes S, Stefanovic D, Chambers SK (2015). Assessing the effectiveness of decision aids for decision making in prostate cancer testing: a systematic review. Psychooncology.

[4] Frosch DL, Bhatnagar V, Tally S, Hamori CJ, Kaplan RM (2008). Internet patient decision support: a randomized controlled trial comparing alternative approaches for men considering prostate cancer screening. Arch Intern Med.

[5] Watts KJ, Meiser B, Wakefield CE, Barratt AL, Howard K, Cheah BC, Mann GJ, Lobb EA, Gaff CL, Patel MI (2014). Online prostate cancer screening decision aid for at-risk men: a randomized trial. Health Psychol.

[6] Salkeld G, Cunich M, Dowie J, Howard K, Patel MI, Mann G, Lipworth W (2016). The Role of Personalised Choice in Decision Support: A Randomized Controlled Trial of an Online Decision Aid for Prostate Cancer Screening. PLoS One.

[7] Ellison GL, Weinrich SP, Lou M, Xu H, Powell IJ, Baquet CR (2008). A randomized trial comparing web-based decision aids on prostate cancer knowledge for African-American men. J Natl Med Assoc.

[8] Elwyn G, Lloyd A, Joseph-Williams N, Cording E, Thomson R, Durand M, Edwards A (2013). Option Grids: shared decision making made easier. Patient Educ Couns.

[9] Scalia P, Durand M, Kremer J, Faber M, Elwyn G (2018). Online, Interactive Option Grid Patient Decision Aids and their Effect on User Preferences. Med Decis Making.

[10] Feldman-Stewart D, Tong C, Brundage MD (2018). Evaluation of a widely available patient decision aid for the treatment of prostate cancer. Patient Educ Couns.

[11] Stacey D, Légaré F, Lewis K, Barry MJ, Bennett CL, Eden KB, Holmes-Rovner M, Llewellyn-Thomas H, Lyddiatt A, Thomson R, Trevena L (2017). Decision aids for people facing health treatment or screening decisions. Cochrane Database Syst Rev.

[12] Légaré F, Kearing S, Clay K, Gagnon S, D'Amours D, Rousseau M, O'Connor A (2010). Are you SURE?: Assessing patient decisional conflict with a 4-item screening test. Can Fam Physician.

[13] Ferron PA, Labrecque M, Rousseau M, Turcotte S, Légaré F (2014). Validation of SURE, a four-item clinical checklist for detecting decisional conflict in patients. Med Decis Making.

[14] Woods-Burnham L, Stiel L, Wilson C, Montgomery S, Durán AM, Ruckle HR, Thompson RA, De LM, Casiano CA (2018). Physician Consultations, Prostate Cancer Knowledge, and PSA Screening of African American Men in the Era of Shared Decision-Making. Am J Mens Health.

[15] Halley MC, Rendle KAS, Gillespie KA, Stanley KM, Frosch DL (2015). An exploratory mixed-methods crossover study comparing DVD- vs. Web-based patient decision support in three conditions: The importance of patient perspectives. Health Expect.

[16] Barry MJ, Wexler RM, Brackett CD, Sepucha KR, Simmons LH, Gerstein BS, Stringfellow VL, Fowler FJ (2015). Responses to a Decision Aid on Prostate Cancer Screening in Primary Care Practices. Am J Prev Med.

[17] (2018). Agency for Healthcare Research and Quality.

[18] Rubel SK, Miller JW, Stephens RL, Xu Y, Scholl LE, Holden EW, Stroud LA, Volk RJ (2010). Testing the effects of a decision aid for prostate cancer screening. J Health Commun.

[19] Joseph-Williams N, Evans R, Edwards A, Newcombe RG, Wright P, Grol R, Elwyn G (2010). Supporting informed decision making online in 20 minutes: an observational web-log study of a PSA test decision aid. J Med Internet Res.

[20] Coulter A, Stilwell D, Kryworuchko J, Mullen PD, Ng CJ, van der Weijden T (2013). A systematic development process for patient decision aids. BMC Med Inform Decis Mak.

[21] Tomko C, Davis KM, Luta G, Krist AH, Woolf SH, Taylor KL (2015). A comparison of web-based versus print-based decision AIDS for prostate cancer screening: participants' evaluation and utilization. J Gen Intern Med.

[22] Kassan EC, Williams RM, Kelly SP, Barry SA, Penek S, Fishman MB, Cole CA, Miller EM, Taylor KL (2012). Men's use of an Internet-based decision aid for prostate cancer screening. J Health Commun.

