# Supplementary material for: Assessing Preference Shift and Effects on Patient Knowledge and Decisional Conflict: Cross-Sectional Study of an Interactive Prostate-Specific Antigen Test Patient Decision Aid
Source: JMIR Cancer. 2018 Nov 21;4(2):e11102. doi: 10.2196/11102 (PMC6282011; doi:10.2196/11102)

Multimedia Appendix 1

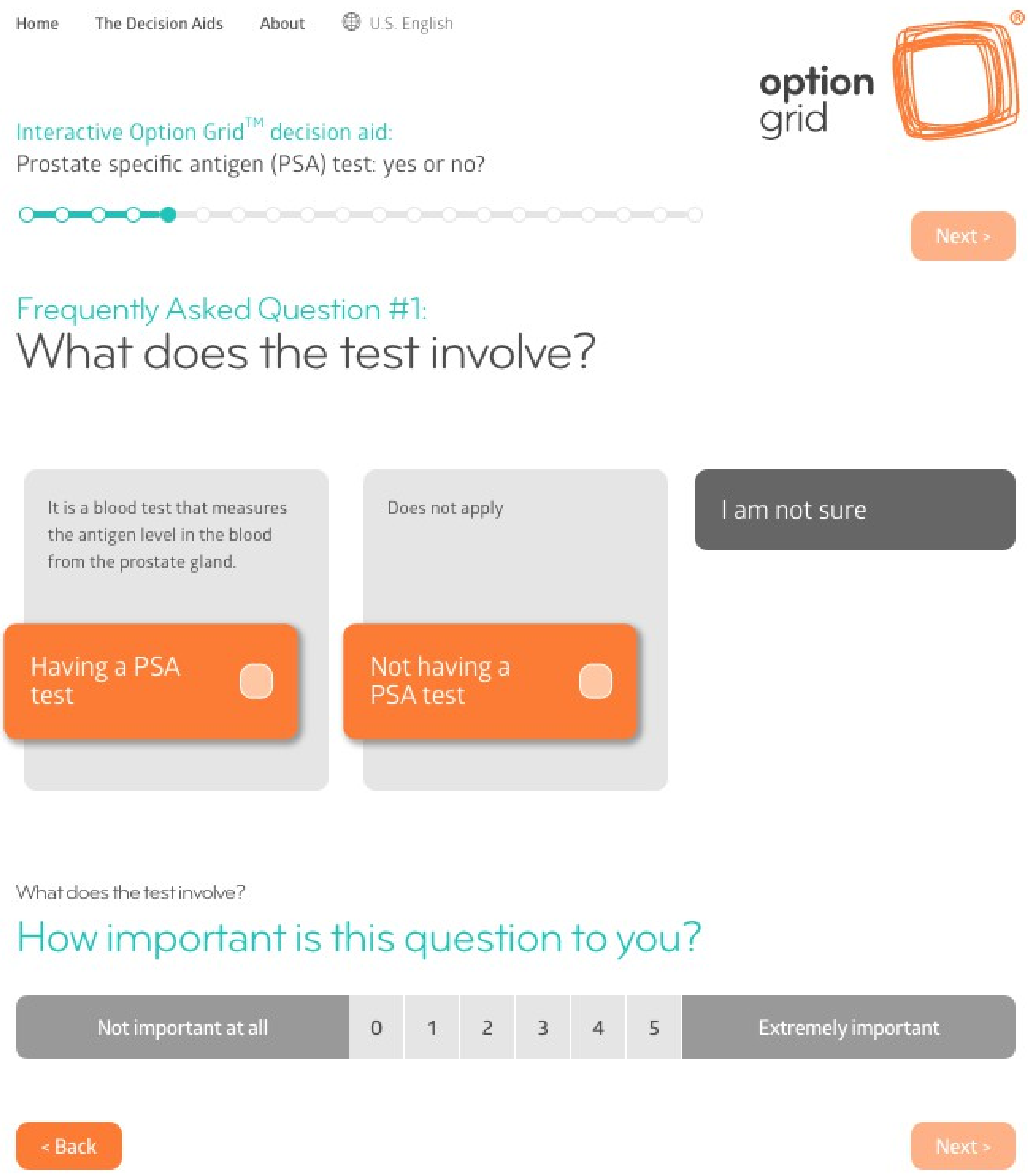

Supplement: Multimedia Appendix 1 [file cancer_v4i2e11102_app1.pdf]
