# Supplementary material for: Assessing Preference Shift and Effects on Patient Knowledge and Decisional Conflict: Cross-Sectional Study of an Interactive Prostate-Specific Antigen Test Patient Decision Aid
Source: JMIR Cancer. 2018 Nov 21;4(2):e11102. doi: 10.2196/11102 (PMC6282011; doi:10.2196/11102)
Supplement: Multimedia Appendix 2 [file cancer_v4i2e11102_app2.pdf]

Multimedia Appendix 2

Self check

What do you already know?

Before you start the interactive journey, let's see what you already know about this topic. You will be able to redo the self-check at the end of the interactive journey to see if your score changes. You aren't being graded!

Question: 1
The PSA test is a blood test.

Your answer:

- [x] True
- [ ] False

Question: 2
Most men who have a high PSA level have prostate cancer.

Your answer:

- [ ] True
- [x] False

Question: 3
A normal PSA test always means that there is no cancer.

Your answer:

- [ ] True
- [x] False

Question: 4
If you do not have a PSA test, there is a small chance that an aggressive prostate cancer might be missed at an early stage.

Your answer:

- [x] True
- [ ] False

Question: 5
The number of men who have a PSA test and die from prostate cancer is similar to the number of men who do not have a PSA test and die from prostate cancer.

Your answer:

- [x] True
- [ ] False
