# Supplementary material for: Assessing Preference Shift and Effects on Patient Knowledge and Decisional Conflict: Cross-Sectional Study of an Interactive Prostate-Specific Antigen Test Patient Decision Aid
Source: JMIR Cancer. 2018 Nov 21;4(2):e11102. doi: 10.2196/11102 (PMC6282011; doi:10.2196/11102)
Supplement: Multimedia Appendix 3 [file cancer_v4i2e11102_app3.pdf]

Multimedia Appendix 3

Question: 1
Do you feel sure about the best choice for you?

Your answer:

- [x] Yes
- [ ] No

Question: 2
Do you know the benefits and risks of each option?

Your answer:

- [x] Yes
- [ ] No

Question: 3
Are you clear about which benefits and risks matter most to you?

Your answer:

- [x] Yes
- [ ] No

Question: 4
Do you have enough support and advice to make a choice?

Your answer:

- [x] Yes
- [ ] No
